# Nephrolithiasis, kidney failure and bone disorders in Dent disease patients with and without CLCN5 mutations

**DOI:** 10.1186/s40064-015-1294-y

**Published:** 2015-09-15

**Authors:** Franca Anglani, Angela D’Angelo, Luisa Maria Bertizzolo, Enrica Tosetto, Monica Ceol, Daniela Cremasco, Luciana Bonfante, Maria Antonietta Addis, Dorella Del Prete, On behalf of the Dent Disease Italian Network

**Affiliations:** Division of Nephrology, Department of Medicine DIMED, Laboratory of Histomorphology and Molecular Biology of the Kidney, University of Padua, Via Giustiniani n° 2, 35128 Padua, Italy; Department of Public Health, Clinical and Molecular Medicine, University of Cagliari, Cagliari, Italy

**Keywords:** Dent disease, CLCN5 gene, OCRL gene, Calcium phosphate metabolism, Nephrolithiasis, Chronic kidney disease

## Abstract

Dent disease (DD) is a rare X-linked recessive renal tubulopathy characterised by low-molecular-weight proteinuria (LMWP), hypercalciuria, nephrocalcinosis and/or nephrolithiasis. DD is caused by mutations in both the CLCN5 and OCRL genes. CLCN5 encodes the electrogenic chloride/proton exchanger ClC-5 which is involved in the tubular reabsorption of albumin and LMW proteins, OCRL encodes the inositol polyphosphate 5-phosphatase, and was initially associated with Lowe syndrome. In approximately 25 % of patients, no CLCN5 and OCRL mutations were detected. The aim of our study was to evaluate whether calcium phosphate metabolism disorders and their clinical complications are differently distributed among DD patients with and without CLCN5 mutations. Sixty-four male subjects were studied and classified into three groups: Group I (with CLCN5 mutations), Group II (without CLCN5 mutations) and Group III (family members with the same CLCN5 mutation). LMWP, hypercalciuria and phosphaturic tubulopathy and the consequent clinical complications nephrocalcinosis, nephrolithiasis, bone disorders, and chronic kidney disease (CKD) were considered present or absent in each patient. We found that the distribution of nephrolithiasis, bone disorders and CKD differs among patients with and without CLCN5 mutations. Only in patients harbouring CLCN5 mutations was age-independent nephrolithiasis associated with hypercalciuria, suggesting that nephrolithiasis is linked to altered proximal tubular function caused by a loss of ClC-5 function, in agreement with ClC-5 KO animal models. Similarly, only in patients harbouring CLCN5 mutations was age-independent kidney failure associated with nephrocalcinosis, suggesting that kidney failure is the consequence of a ClC-5 dysfunction, as in ClC-5 KO animal models. Bone disorders are a relevant feature of DD phenotype, as patients were mainly young males and this complication occurred independently of age. The triad of symptoms, LMWP, hypercalciuria, and nephrocalcinosis, was present in almost all patients with CLCN5 mutations but not in those without CLCN5 mutations. This lack of homogeneity of clinical manifestations suggests that the difference in phenotypes between the two groups might reflect different pathophysiological mechanisms, probably depending on the diverse genes involved. Overall, our results might suggest that in patients without CLCN5 mutations several genes instead of the prospected third DD underpin patients’ phenotypes.

## Background

Dent disease (DD) is a rare X-linked recessive renal tubulopathy characterised by low-molecular-weight proteinuria (LMWP) and is often associated with hypercalciuria, nephrocalcinosis and/or nephrolithiasis. Additional tubular defects, such as hyperphosphaturia, glycosuria and aminoaciduria, may also be observed (Devuyst and Thakker 2010). Progression to chronic kidney disease (CKD) and renal failure occurs between the 3rd and 5th decades of life in 30–80 % of male patients (Wrong et al. 1994; Lloyd et al. 1997; Thakker 2000). Signs generally manifest in childhood or in early adult life. The presentation of DD appears variable: a universal feature is proteinuria, but other presenting features in children and adolescents include nephrocalcinosis and hypercalciuria, with or without nephrolithiasis, although the relationship between nephrocalcinosis and stone formation remains unclear. Hypophosphataemia and bone disorders (BDs) have also been observed but appear to be a less prominent feature of the phenotype than originally thought. In adulthood, the disease is suspected in most cases due to the presence of nephrocalcinosis/nephrolithiasis or unexplained CKD in subjects with hypercalciuria or in the context of familial cases of CKD and/or nephrolithiasis (Edvardsson et al. 2013; Ferraro et al. 2013).

DD is caused by mutations in the CLCN5 gene, which encodes the electrogenic chloride/proton exchanger ClC-5 (this condition is now called Dent disease 1, OMIM 300009) (DD1). In the human kidney, ClC-5 is primarily expressed in proximal tubular cells (PTCs), predominantly located in the subapical endosomes, which are involved in the endocytic reabsorption of albumin and LMW proteins (Jouret et al. 2004; Picollo and Pusch 2005). To date, more than 200 different mutations have been reported in the CLCN5 gene (Mansour-Hendili et al. 2015). However, in approximately 40 % of patients with the classic DD phenotype, no CLCN5 gene mutations have been detected. Mutations in the OCRL gene, encoding the inositol polyphosphate 5-phosphatase, initially associated with Lowe syndrome, account for 15 % of the patients now classified as having Dent disease 2 (DD2) (OMIM 300555) (Hoopes et al. 2004, 2005). The spectrum of symptoms in DD2 can range from apparent exclusive kidney manifestations to the involvement of other tissues, notably brain and muscle, as in Lowe syndrome (Hichri et al. 2011). Approximately 25 % of patients, however, do not harbour mutations in CLCN5 or OCRL (Hoopes et al. 2004, 2005; Tosetto et al. 2009a, b). Genotype–phenotype correlations have yet to be established. Whereas ClC-5 knock-out (KO) models recapitulated the major features of DD (Silva et al. 2003; Sayer et al. 2004), the OCRL1 KO mice did not (Jänne et al. 1998). For this reason, the pathogenic mechanisms through which the loss of OCRL1 function leads to DD are not yet deeply understood (Mehta et al. 2014).

The aim of our study was to evaluate whether calcium phosphate metabolism disorders and their clinical complications are differently distributed among DD patients with and without CLCN5 mutations.

## Results

On the basis of the genetic analysis and recruitment criteria (unrelated or familial case), the patients were classified into three groups: Group I, 41 patients (20 adults and 21 children) with CLCN5 mutations (CLCN5+); Group II, 17 patients (8 adults and 9 children) without CLCN5 mutations (CLCN5−); Group III, the 6 family members (4 adults and 2 children) who carried the same CLCN5 mutation (CLCN5+ family).

The frequencies of the seven clinical signs considered were calculated, and their absolute and percentage values in the three groups are shown in Table 1. In descending order of frequency, these signs were LMWP 98.4 %, hypercalciuria 85.9 %, nephrocalcinosis 71.9 %, phosphaturic tubulopathy 40.6 %, bone disorders 34.4 %, nephrolithiasis 29.7 %, and CKD 14.1 %. However, the frequencies were differed somewhat among the three groups, except for LMWP, which was present in 100 % of the cases in Groups I and II, given that it was required for recruitment, and in 83.3 % of the subjects in Group III (5/6), who were recruited because of family history and genetic positivity. Nephrolithiasis was quite rare in our patients, being detected in only 26.8 % of the patients in Group I and in 29.4 % of Group II. BDs were found in 41.5 % of the patients in Group I, in 29.4 % in Group II, and in none of the patients in Group III. Only 2 out of 17 patients presenting with BDs of Group I had a T-score <−2.5. CKD was observed in 12.2 % of the Group I patients, in 23.5 % of Group II, and in none of the Group III patients. Positive family history of nephropathy was equally distributed in the first two groups and specifically in 48.3 % of the 58 patients belonging to the combined groups.

**Table 1:** Absolute and percentage values of the seven clinical signs considered

	Total patients		Group I		Group II		Group III	
	Number	%	Number	%	Number	%	Number	%
Males	64		41		17		6	
Median age	16 ± 12		14 ± 9		18 ± 16		27 ± 16	
Range	1–58 years		1–43 years		2–58 years		2–46 years	
LMWP	63	98.4	41	100	17	100	5	83.3
Nephrocalcinosis	46	71.9	36	87.8*^§^	7	41.2	3	50
Nepholithiasis	19	29.7	11	26.8	5	29.4	3	50
Hypercalciuria	55	85.9	40	97.6**^§§^	12	70.6	3	50
Phosphaturic tubulopathy	26	40.6	13	31.7	9	52.9	4	66.7
Bone disorders	22	34.4	17	41.5	5	29.4	0	0
Kidney failure	9	14.1	5	12.2	4	23.5	0	0

Group I versus Group II: * p < 0.001, ** p < 0.01. Group I versus Group III: ^§^ p = 0.05, ^§§^ p < 0.01

To evaluate the transversal distribution of calcium phosphate metabolism disorders and their complications, we compared the frequencies of each clinical sign in the three groups. A comparison among groups revealed that nephrocalcinosis (p < 0.001) and hypercalciuria (p < 0.01) were more frequent in Group I than in Group II and more frequent in Group I than III (p = 0.05 and p < 0.01, respectively).

No association was found between age and the presence of the various signs considered in either the CLCN5^+^ or CLCN5^−^ group. Nephrolithiasis, however, was significantly (p = 0.011) more frequent in adults (12/28) than in children (3/30), but the median age of adult patients with nephrolithiasis was significantly lower in CLCN5^+^ than in CLCN5^−^ group (23 vs. 42 p = 0.012). In contrast, in the CLCN5^+^ family, nephrolithiasis was present only in the three adults aged 38–46 years.

Associations between two clinical signs within a single group were found between nephrocalcinosis and nephrolithiasis (p < 0.0001) and between hypercalciuria and nephrolithiasis (p < 0.0001) in Group I. Whereas the association between nephrocalcinosis and CKD was significant in Group I (p < 0.0001), it was not in Group II, whose patients presented with CKD more frequently (23.5 vs. 12.2 %). BDs were not associated with any of the other parameters studied in Groups I and II, and they were not present in any of the patients in Group III.

## Discussion

Although we found a low number of significant differences, the distribution of the clinical signs among the three groups of patients seems to reveal different renal phenotypes. Whereas in Group I the predominant phenotype was characterised by hypercalciuria and nephrocalcinosis, in Group II and III, it was better represented by a phosphaturic tubulopathy.

Hypercalciuria and nephrocalcinosis, which together with LMWP represent the main clinical manifestations in our DD1 patients and patients in the literature, were not frequent in CLCN5^−^ patients. Particularly, nephrocalcinosis had a low frequency that was similar to that observed by Bokenkamp et al. (2009) in DD patients harbouring OCRL mutations. Claverie-Martín et al. (2011) reported a frequency of rickets and osteomalacia in DD1 patients similar to ours. CKD is reported to occur in more than two-thirds of DD1-affected males, a frequency much higher than ours, but we classified our patients as having CKD only when CKD was clearly evident and stable (CKD stage 3). In our DD1 patients, nephrolithiasis was not as frequent as nephrocalcinosis (26.8 vs. 87.8 %, respectively). Also the meta-analysis of Claverie-Martín et al. (2011) reported that nephrolithiasis occurs in DD1 patients less frequent than nephrocalcinosis (41 vs. 76 %).

Although the absence of metabolic abnormalities linked to calcium phosphate metabolism in patients with relapsing or recurring episodes of idiopathic calcium nephrolithiasis is rather frequent—higher than 40 % (personal unpublished data)—nephrolithiasis in DD patients should, by definition, always be secondary to tubulopathy. In fact, kidney stones without metabolic abnormalities were identified sporadically in all three groups. However, hypercalciuria, the most important metabolic abnormality associated with Dent disease (Devuyst and Thakker 2010), was associated with concomitant nephrolithiasis only in CLCN5^+^ patients.

A family history of nephropathy was present in approximately half of our patients, and it was equally frequent in Groups I and II, further confirming the importance of genetic background in Group II. A family history of nephrolithiasis is highly variable in stone formers (Gambaro et al. 2004). Kidney stones are a heterogeneous disease, but our patients were selected based on having a genetic disease, and this is why positive family history was so frequent.

Subdividing patients into CLCN5-positive and -negative groups allowed us to evaluate whether different genetic backgrounds were linked to different clinical and metabolic profiles. In fact, whereas in patients with CLCN5 mutations the pathogenesis of DD was linked to modifications in the chloride/proton exchanger ClC-5, with consequent alterations in the PTC endocytic processes, in CLCN5 and OCRL-negative patients, the pathogenesis of the disease remained unclear.

The family group was separated from the patients in Group I to verify whether the disease’s clinical aspects in the family with the same CLCN5 mutation overlapped with those found in the forty-one unrelated patients with different CLCN5 mutations and, if the phenotype expression was different, to specify how. In fact, only one of the family members presented with manifest signs of the disease, whereas none of the other six were suspected of having DD despite their ages and did not exhibit clinical signs that were consistent with the DD phenotype.

The defining characteristic of DD is the presence of LMWP. In our patients, only one out of 64 did not present with LMWP. This patient was a 12-year-old male, belonging to the family group, who at the time of recruitment did not show any of the clinical signs considered. To our knowledge, this is the second case reported of a DD patient not presenting with LMWP (Scheinman et al. 2000). Of note, this subject subsequently and gradually manifested higher LMWP, which even exceeded 5 times the initial value that we measured. His delayed manifestation of LMWP and of other disease symptoms strongly supports the presence of modifier genes modulating disease expression. In line with this hypothesis is the fact that in this family none of the heterozygous females presented with LMWP.

Nephrocalcinosis, present at an elevated frequency in our population and significantly higher in CLCN5^+^ patients with respect to the CLCN5^−^ subjects, was absent in only four of the CLCN5^+^ patients, three of whom were of preschool or school age and might presumably develop this clinical sign at a later date. Nephrocalcinosis was associated with hypercalciuria in Group I, and both were significantly more frequent than in Group II. These data suggest that the different genotypes—mutations of CLCN5 in Group I and of other genes (different from CLCN5 and OCRL genes) in Group II—may be responsible for the different frequencies of intraparenchymal calcium deposition. Within the family group, however, the presence of nephrocalcinosis was significantly lower compared to Group I, although the underlying pathogenic mechanisms should be the same. Again, it could be rationally hypothesised that modifying genes contributed to this lighter phenotypic expression.

Unlike the relatively low but numerically similar frequency of nephrolithiasis and nephrocalcinosis in Groups II and III, in Group I, the presence of nephrolithiasis was much lower than that of nephrocalcinosis, although in this group, nephrolithiasis, when present, was practically always associated with nephrocalcinosis. Thus, nephrolithiasis appears to be a direct consequence of nephrocalcinosis and is also accompanied by hypercalciuria. Therefore, nephrolithiasis was linked to hypercalciuria in Group I as an expression of the PTC damage and a consequence of the dysfunction of the chloride/proton exchanger CIC-5. Our results, then, may confirm in humans what has been shown in different ClC-5 KO animal models and in vitro studies (Wang et al. 2000; Silva et al. 2003; Sayer et al. 2004).

In contrast to Group I, in Groups II and III, nephrolithiasis seemed to be an independent manifestation of nephrocalcinosis, mostly being present in adults. Thus, in the majority of CLCN5^−^ patients and in the CLCN5^+^ family, nephrolithiasis was associated neither with nephrocalcinosis nor with hypercalciuria. According to our data, in these patients, nephrolithiasis appeared to follow a different pathogenic pathway with respect to the patients of Group I.

Hypercalciuria is frequent in patients affected by DD1 and is an important diagnostic parameter. This relation suggests a substantially proximal tubular dysfunction, either through an indirect mechanism mediated by vitamin D (Silva et al. 2003) or through the derangement of calcium reabsorption in the PTCs by endocytosis (Friedman 1999; Devuyst et al. 1999) both being due to the loss of CIC-5 function. The mechanism by which hypercalciuria arises in CLCN5^−^ patients is unknown, but the significantly lower frequency with which it was found in our CLCN5^−^ patients with respect to the CLCN5^+^ patients suggests that the endocytosis-mediated proximal calcium reabsorption is intact.

In the entire sample of DD patients, BDs were not associated with any of the parameters considered, but they were nevertheless observed in more than 30 % of our patients. This is undoubtedly a relevant finding considering that all patients were males with a median age of 16 and that this complication occurred independently of age. Furthermore, BDs were neither significantly different between the CLCN5^+^ and CLCN5^−^ patients nor significantly associated with the presence of hypercalciuria. It is noteworthy, however, that in children with CLCN5 mutations the clinical finding of rickets were present in 9 out of 21, almost two time more frequent than in children without CLCN5 mutations where only 2 out of 9 presented with rickets. In the family, the total absence of BDs suggests a shared genetic background protecting against bone demineralisation. The fact that the heterozygous females of the family had even higher bone density than normal reference parameters may support this hypothesis (data not shown).

Similarly, CKD was absent in all 6 members of the family, even in those with nephrocalcinosis and/or nephrolithiasis who were old enough that the renal dysfunction could have already evolved into CKD. This observation is notable in consideration of the existence of a one-year-old infant with CKD in Group I. Once again, important modifier genes may have influenced disease expression in the CLCN5^+^ family.

That DD is a hereditary form of nephrolithiasis associated with CKD is well known (Edvardsson et al. 2013). Two large families were described in the 1990s by Frymoyer et al. (1991) and Wrong et al. (1994). These authors found that nephrolithiasis and CKD segregated as an X-linked recessive trait. The pathogenic mechanism leading to CKD in DD patients, however, is not completely understood. The most popular hypothesis is that renal calcium deposits lead to CKD through a chronic interstitial inflammation developing into fibrosis (Cebotaru et al. 2005). Our findings confirm this hypothesis only in part. Supporting this hypothesis are the data from Group I, in which all subjects with CKD had concomitant nephrocalcinosis, and the occurrence of CKD was not age-related. At odds with the hypothesis is the finding that the majority of patients with CKD belonged to the CLCN5^−^ group, in which this complication was not associated with nephrocalcinosis.

On the basis of these findings, an alternative hypothesis can be advanced: the genes involved in the development of the DD phenotype in the CLCN5^−^ patients cause CKD through mechanisms other than those in the CLCN5^+^ patients, where the associations between hypercalciuria and nephrocalcinosis and between nephrocalcinosis and CKD seem to indicate the direct involvement of the ClC-5 alteration in the pathogenesis of nephrocalcinosis and subsequent CKD, as shown in animal models (Cebotaru et al. 2005).

## Conclusions

Our data indicate that the distribution of seven clinical signs, particularly of the 4 main complications that typically characterise DD, is different among the two genotypically different groups. This fact might indicate that although the clinical manifestations appear rather similar, the pathogenesis is different, probably depending on the diverse genes involved.

In fact, the triad of symptoms, LMWP, hypercalciuria, and nephrocalcinosis, was present in almost all patients of Group I, indicating that the loss of ClC-5 function was the cause of clinical manifestations independently of allele heterogeneity, thus mimicking the picture shown in the ClC-5 KO mouse models. This situation might not be true for patients of Group II, who should harbour mutations in different gene/genes. This lack of homogeneity of clinical manifestations might suggest that several genes underpin our patients’ phenotypes instead of the prospected third DD gene.

Clinical symptoms in the CLCN5^+^ family members are quite variable and more similar to those of Group II, although a common CLCN5 mutation segregates in the family’s affected members. To explain this atypical picture, the presence of a shared genetic background (modifier genes) modifying the DD1 phenotypic manifestations should be strongly suspected.

## Patients and methods

### Patients

Sixty-four male subjects were evaluated: 58 unrelated patients came to our attention because of the suspicion of DD, and 6 were recruited because they were relatives of one of these patients and were positive for a CLCN5 mutation. The latter six patients did not come to our attention because of evident DD symptomatology but because they were related to one of the 58 patients who was the only one in the family to have shown a DD phenotype.

In this study, the DD phenotype was defined as a clinical picture characterised by an association of LMWP with hypercalciuria and/or at least one of the following: nephrocalcinosis, nephrolithiasis, phosphaturic tubulopathy, BDs, CKD as well as family history of nephropathy (Tosetto et al. 2006).

The nephrology centres across Italy that followed these patients provided us with clinical and laboratory data. Based on the analysis of these data, we utilised three clinical/metabolic signs (LMWP, hypercalciuria and phosphaturic tubulopathy) and four clinical complications (nephrolithiasis, nephrocalcinosis, BDs, and CKD) that, together with family history of nephropathy, were recorded as present or absent in each patient. The mean age of patients was 16 ± 12 years (1–58 years). Thirty-two were children, age <14 years. All the 64 patients were males, because the disease is known to be transmitted as an X-linked recessive trait. However, in almost all patients evidence of an X-linked disease transmission was absent. The study was conducted according to the principles of the Helsinki Declaration and was approved by the local IRB.

### Definitions

LMWP was qualitatively evaluated as 24-h urinary increased excretion of β2-microglobulin (at least five times higher than the normal value of <300 µg/day), although in some cases the increase was lower than that typical in DD.

Hypercalciuria was defined as fasting and absolute (urinary calcium excretion >300 mg/day in adults or >4 mg per kg of bodyweight in children per day). Phosphaturic tubulopathy was defined as persistent hypophosphatemia in the presence of a decrease tubular reabsorption of phosphate (TRP <80 %) and/or reduced renal threshold phosphate concentration (TmPO_4_/GFR).

Nephrolithiasis was attributed if a stone had been passed or removed or shown in the urinary tract by X-ray or ultrasound imaging. Nephrocalcinosis was defined as diffuse, fine, renal parenchyma calcification on X-ray and/or ultrasound examinations.

Bone disorders are defined as the presence of rickets or bone demineralisation. Rickets were evaluated in children during the physical and skeletal radiological examinations and bone demineralisation in adults by measuring bone density of the lumbar spine and femoral neck using dual energy X-ray absorptiometry (DEXA) (T-score <−1.5 SD).

CKD in both children and adults was attributed when creatinine clearance (CCr) was <60 ml/min/1.73 m^2^ (CKD stage 3). In all our patients parathyroid hormone (normal range 10–65 ng/l in adults and 10–55 ng/l in children) and serum calcium were normal. None of our patients had CCr <30 ml/min/1.73 m^2^.

Family history was considered positive when a nephropathy—most frequently nephrolithiasis or CKD—was present in one or more family members.

Clinical diagnosis of DD was considered “probable” when the patient presented with LMWP and hypercalciuria in association with at least one of the other signs indicated above and “possible” when the patient presented with LMWP with at least one of the other signs and in the absence of hypercalciuria (Tosetto et al. 2006).

Molecular analysis, based on the assessment of CLCN5 and OCRL mutations (Tosetto et al. 2006, 2009a) detected 37 different CLCN5 mutations and confirmed a DD1 clinical diagnosis in 41 out of the 58 suspected DD patients (for molecular genetic details see refs. Tosetto et al. 2006, 2009b). Seventeen patients did not carry any CLCN5 mutation. Mutational screening of OCRL gene did not reveal any mutation. Cascade genetic screening was conducted in 11 male subjects of the family because they were sons of the heterozygous females, and 6 resulted to carry the same G260V CLCN5 mutation as the proband (Anglani et al. 2006).

### Statistics

The McNemar test was used to evaluate the significance with which two clinical signs in the same group were associated. Fisher’s exact test was used for small samples not numerically adequate for the χ^2^ test. The χ^2^ test was used to assess the variation in the frequency of the same clinical sign in two different groups to indicate the non-randomness of the difference and, therefore, the lack of homogeneity of the two classes being examined. The χ^2^ test was also applied to evaluate the association between age and the presence of various clinical signs in each of the three groups considered.
